# Statistical evaluation of transcriptomic data generated using the Affymetrix one-cycle, two-cycle and IVT-Express RNA labelling protocols with the *Arabidopsis *ATH1 microarray

**DOI:** 10.1186/1746-4811-6-9

**Published:** 2010-03-15

**Authors:** Tara J Holman, Michael H Wilson, Kim Kenobi, Ian L Dryden, T Charlie Hodgman, Andrew TA Wood, Michael J Holdsworth

**Affiliations:** 1 Centre for Plant Integrative Biology, University of Nottingham, Nottingham, LE12 5RD, UK; 2 School of Mathematical Sciences, University of Nottingham, Nottingham, NG7 2RD, UK; 3 Department of Plant and Crop Sciences, School of Biosciences, University of Nottingham, Nottingham, LE12 5RD, UK

## Abstract

**Background:**

Microarrays are a powerful tool used for the determination of global RNA expression. There is an increasing requirement to focus on profiling gene expression in tissues where it is difficult to obtain large quantities of material, for example individual tissues within organs such as the root, or individual isolated cells. From such samples, it is difficult to produce the amount of RNA required for labelling and hybridisation in microarray experiments, thus a process of amplification is usually adopted. Despite the increasing use of two-cycle amplification for transcriptomic analyses on the Affymetrix ATH1 array, there has been no report investigating any potential bias in gene representation that may occur as a result.

**Results:**

Here we compare transcriptomic data generated using Affymetrix one-cycle (standard labelling protocol), two-cycle (small-sample protocol) and IVT-Express protocols with the Affymetrix ATH1 array using Arabidopsis root samples. Results obtained with each protocol are broadly similar. However, we show that there are 35 probe sets (of a total of 22810) that are misrepresented in the two-cycle data sets. Of these, 33 probe sets were classed as mis-amplified when comparisons of two independent publicly available data sets were undertaken.

**Conclusions:**

Given the unreliable nature of the highlighted probes, we caution against using data associated with the corresponding genes in analyses involving transcriptomic data generated with two-cycle amplification protocols. We have shown that the Affymetrix IVT-E labelling protocol produces data with less associated bias than the two-cycle protocol, and as such, would recommend this kit for new experiments that involve small samples.

## Background

Over the past fifteen years microarray technology has revolutionised molecular biology. Where once determination of quantitative expression levels of genes involved performing Northern blot analyses, microarrays have made possible the investigation of the expression level of thousands of genes in a single experiment [1,2]. In recent years the cost of performing microarray experiments has decreased dramatically, as has the quantity of RNA required. Early experiments to analyse global gene expression profiles in *Arabidopsis *research involved using large quantities of tissue in order to generate the required amount of RNA. For example, Schaffer *et al*. [3] required 1 g of fresh tissue to extract 100 μg RNA to use with a cDNA spotted array; Affymetrix currently recommends using as little as 50 ng total RNA for labelling with the ATH1 expression array http://www.affymetrix.com. The reduction in the quantity of material required has led to an increasing focus on the analysis of less abundant tissues, subsets of cells or even individual cells, isolated using techniques such as laser microdissection and fluorescence-activated cell sorting [4-10]. At present, it is not possible to extract sufficient RNA from micro-dissected samples for transcriptomic analysis, so various methods of RNA amplification have been developed and implemented [11-14].

For transcriptomic analyses, extracted RNA must be labelled prior to hybridisation to the array. Standard protocols (one-cycle labelling), such as the Affymetrix One-Cycle Eukaryotic Target Labelling Assay and a new protocol suitable for small samples, the Affymetrix GeneChip 3' IVT-Express Kit (IVT-E), use oligo(dT) primers with a T7 recognition site to reverse transcribe mRNA to cDNA. DNA polymerase is used for the production of double stranded cDNA. T7 RNA polymerase, in the presence of biotinylated nucleotides, is used for *in vitro *transcription (IVT) of biotinylated cRNA, which is hybridised to the array. Small sample protocols follow a similar methodology, but the initial IVT step uses unlabeled nucleotides. Using random primers, the mRNA is reverse transcribed, followed by amplification using the oligo(dT)-T7 primer. The second IVT uses T7 RNA polymerase to incorporate biotinylated nucleotides into the mRNA.

Several commercial kits are available for producing two-cycle amplified samples for hybridisation to microarrays, for example the Two-Cycle Eukaryotic Target Labelling Assay (Affymetrix, High Wycombe, UK), the Microarray Target Amplification Kit (Roche Diagnostics Ltd. Burgess Hill, UK), the MessageAmp aRNA Amplification Kit (Ambion, Texas, USA), and the FL-Ovation Biotin System (Nugen Technologies, California, USA). Results produced using various commercial kits have been analysed using human samples, and it was shown that the Affymetrix amplification system showed the highest correlation to the standard one-cycle protocol [15].

The Affymetrix ATH1 microarray (Affymetrix UK Ltd., High Wycombe, UK) is frequently used for Arabidopsis transcriptomic analyses. The array consists of 22759 gene-specific probe sets, each containing eleven perfect match (PM) and eleven mis-matched (MM) probes (twenty-five base oligonucleotides hybridised to a glass slide). PM probes are complementary to the mRNA sequence; MM probes differ from the PM probes only at nucleotide thirteen, where the base is swapped to its complementary partner (e.g. C to G, A to T etc.). The array represents 22543 individual *Arabidopsis *loci (The Arabidopsis Information Resource release 8 (TAIR 8)), with some loci represented by more than one probe set. Following recent updates to the *Arabidopsis *annotated genome, it has been found that up to 10,000 loci may not be represented on the array (TAIR8), thus the ATH1 array has genomic coverage of approximately 70%.

Two-cycle sample labelling protocols have been used with the Affymetrix *Arabidopsis *ATH1 microarray in many experiments [4,16-20]. Whilst an evaluation of the effects of amplification has been carried out for plant samples with two-colour arrays [21], a direct comparison of one- and two-cycle labelled samples has not been published for the Affymetrix ATH1 microarray. This is despite investigations showing that sample amplification causes errors when using the Affymetrix human HG-U133A array [22,23].

Here we describe the analysis of gene expression data generated using Affymetrix one- and two-cycle and IVT-E labelling protocols with the ATH1 microarray. We show that all protocols yield similar results in terms of relative levels of gene expression. There is, however, a subset of genes that are consistently mis-represented by the two-cycle process. We show that two-cycle labelling is an acceptable method for generating samples for use with the Affymetrix ATH1 microarray in situations when enough material cannot reasonably be generated otherwise. However, data relating to the loci highlighted in this report should be treated with caution. Based on the analyses presented here, we recommend the use of the Affymetrix IVT-E labelling protocol for small samples.

## Results

RNA was isolated from 7-day-old Arabidopsis wild-type (Col-0) roots dissected into two sections, the meristem (MS) and elongation zone (EZ). The MS, the region of the root where cell division occurs, is approximately 350 μm in length and extends from the tip of the root to the top of the lateral root cap. The EZ extends from the top of the MS to the first root hair bulge, and is where the cells are rapidly expanding in length. The EZ is approximately 850 μm in length. An Arabidopsis root is around 100 μm in diameter after 7-days development, thus around 365 roots would need to be dissected in to obtain 1 mm^3 ^of material.

For these experiments, three biological replicates from separate pools of seed were used, resulting in six RNA samples (three MS replicates and three EZ replicates). An aliquot of RNA of each sample was diluted to a concentration of 50 ng μl^-1 ^and labelled using both the Affymetrix two-cycle labelling protocol and the Affymetrix IVT-E protocol. One microgram of total RNA was labelled using the Affymetrix one-cycle labelling protocol. A total of eighteen data sets were produced from the original six RNA extractions, with three replicates each of MS one-cycle, MS two-cycle, MS IVT-E, EZ one-cycle, EZ two-cycle and EZ IVT-E. These labelled samples were hybridised to the Affymetrix ATH1 gene expression array.

### Correlation between replicates and between protocols

**Figure 1:**
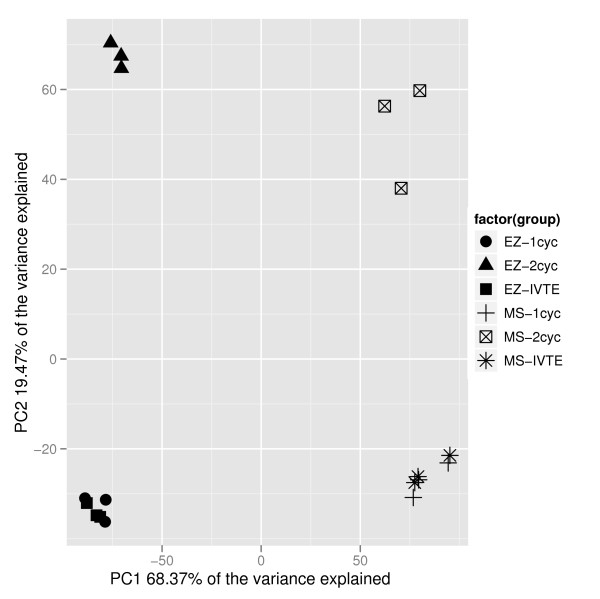
**Principal component analysis of the transcriptomic samples**. Principal component analysis of the eighteen samples (three replicates each of MS one-cycle (MS-1cyc), MS two-cycle (MS-2cyc), MS IVT-E (MS-IVTE), EZ one-cycle (EZ-1cyc), EZ two-cycle (EZ-2cyc) and EZ IVT-E (EZ-IVTE)). Samples clustered closely together have a high level of similarity in expression levels; samples spread far apart have more divergent expression profiles. One-cycle and IVT-E samples cluster nearby each other for each tissue, whilst the two-cycle data sets are divergent.

To investigate the reproducibility of the microarray data, replicates were analysed pair-wise for consistency. The R^2 ^value of log_2 _data ranged from 0.973 to 0.993, showing a large degree of correlation between replicates (Additional file 1). Principal Component Analysis (PCA) was used to show the global differences between the samples. The resulting plot showed that the three replicates from each labelling technique were highly similar, and that the IVT-E and one-cycle data sets were more similar to each other than either were to the two-cycle data (for both MS and EZ tissues) (Figure 1). The first principal component represented the differences between the MS and EZ tissues, whereas the second principal component represents differences between the one-cycle/IVT-E versus two-cycle protocols. To further investigate the differences in labelling protocol, R^2 ^values of pair-wise comparisons of the three protocols for each of the replicates showed that the two-cycle protocol produced data that was less similar to the one-cycle and IVT-E data sets (R^2 ^values between 0.9312 and 0.9532), than the one-cycle and IVT-E data sets were to each other (R^2 ^values between 0.9751 and 0.981) (Table 1). This implies that there is a reduced linearity in the relationship between the low RNA concentration samples and total RNA samples when the IVT-E protocol is used rather than the two-cycle protocol.

**Table 1:** Statistical comparison of the biological replicates

Region	Comparison	Rep 1	Rep 2	Rep 3	Average
MS	1-cycle vs. 2-cycle	0.948	0.948	0.938	**0.945**
MS	2-cycle vs. IVT-E	0.947	0.953	0.941	**0.947**
MS	1-cycle vs. IVT-E	0.980	0.980	0.977	**0.979**

EZ	1-cycle vs. 2-cycle	0.939	0.934	0.935	**0.936**
EZ	2-cycle vs. IVT-E	0.936	0.932	0.931	**0.933**
EZ	1-cycle vs. IVT-E	0.981	0.981	0.975	**0.979**

MS	Pairwise one-cycle	-	-	-	**0.985**
MS	Pairwise two-cycle	-	-	-	**0.978**
MS	Pairwise IVT-E	-	-	-	**0.981**

EZ	Pairwise one-cycle	-	-	-	**0.991**
EZ	Pairwise two-cycle	-	-	-	**0.982**
EZ	Pairwise IVT-E	-	-	-	**0.989**

The top half of the table shows the R^2 ^values for the comparisons for each of the three biological replicates (Rep 1-3) of meristem (MS) and elongation zone (EZ) tissues with the three labelling protocols. The bottom half of the table shows the average R^2 ^values of the pairwise replicate comparisons for each labelling protocol (i.e. the average of rep1 vs. rep2, rep1 vs. rep3 and rep 2 vs. rep3).

### 5' or 3' bias caused by amplification

To determine if there was a differential bias in hybridisation for probes towards either the 5' or 3' ends of the gene caused by the different labelling protocols, the level of hybridisation at individual probes on the array were examined. It was expected that incomplete IVT would cause a 3' probe bias (increased hybridisation levels at probes nearest the 3' end of the gene), and a large amount of cRNA degradation would lead to a bias towards 5' probe expression.

The eleven PM probes in a probe-set were numbered from 1 (nearest the 5' end of the mRNA) to 11 (nearest the 3' end of the mRNA). For each probe-set, the median of the log_2 _expression levels on probes 1 to 5 and the median of the log_2 _expression levels on probes 7 to 11 were calculated. The difference between these values gives a measure of the degree of 5' bias for that gene, with positive numbers indicating 5' bias and negative numbers indicating 3' bias.

**Figure 2:**
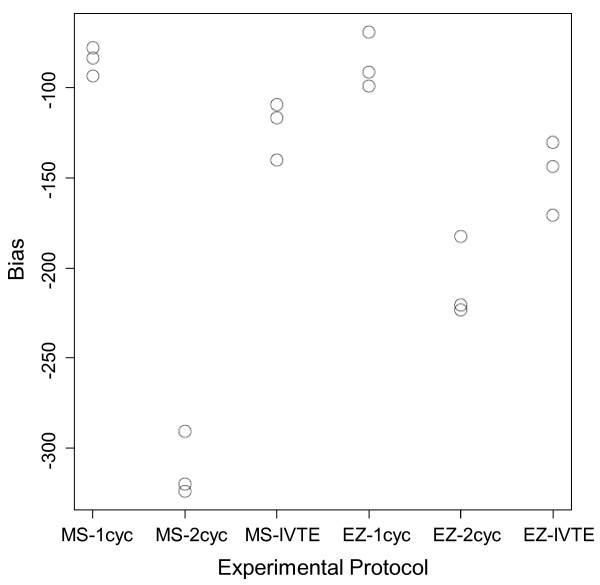
**Mean bias statistics for the three protocols**. Mean bias statistics for the three protocols across the MS and EZ zones and three replicates. More negative numbers indicate increased bias.

For all of the labelling protocols, there is evidence of some degree of 3' bias (i.e. the averages of the distributions are to the left of zero), and this 3' bias was more pronounced in data from the two-cycle protocol for both the MS and EZ tissues (Figure 2). The values corresponding to the two-cycle protocol are more negative than the one-cycle and IVT-E mean scores, indicating an increased level of bias.

To further investigate the phenomenon of increased 3' bias in the two-cycle data, we used quantile plots. Analysis of the results showed that the two-cycle protocol introduced a marked increase in 3' bias in comparison to the one-cycle and IVT-E protocols (Additional file 2). There was a slight increase in 3' bias in the IVT-E data in comparison to the one-cycle data, but the difference was much less than for the two-cycle data.

### Differentially expressed loci between the labelling protocols

Linear models of log_2 _expression levels were fitted pair-wise for data generated using the one-cycle, two-cycle and IVT-E protocols. The studentised residuals were used to assess which of the loci showed differential expression between two protocols. For such a large number of degrees of freedom, the studentised residuals follow a standard Normal distribution. We set a threshold of 3.5 for the residuals (corresponding to 3.5 standard deviations from the mean, and covering 99.95% of data points), classifying those loci with a residual greater than 3.5 as over-amplified and those with a residual less than -3.5 as under-amplified.

As a consequence of the data having a standard Normal distribution, we would expect only 10 of the 22543 loci to have a studentised residual with an absolute value greater than 3.5. We observe in the order of 100 loci with absolute residual values greater than 3.5 for each of the tissues (Table 2 and Additional file 3).

**Table 2:** The number of over- and under-amplified probes

		Number over-amplified loci			Number under-amplified loci		
Region	Comparison	Rep 1	Rep 2	Rep 3	Rep 1	Rep 2	Rep 3
MS	1-cyc vs. 2-cyc	35	20	32	143	148	127
MS	IVT-E vs. 2-cyc	37	16	32	87	127	94

EZ	1-cyc vs. 2-cyc	39	46	39	119	107	112
EZ	IVT-E vs. 2-cyc	38	42	35	101	85	93

The number of over- and under-amplified loci for pair-wise comparisons of two-cycle vs. one-cycle and IVT-E protocols for the three replicates (Rep 1-3).

To define a probe-set as 'mis-amplified' we used the criterion that it must be either classed as over- or under-amplified in at least two of the three replicates, and in both the one-cycle vs. two-cycle and IVT-E vs. two-cycle lists. We found 9 loci that were over-amplified by the two-cycle protocol, and 26 genes under-amplified by the two-cycle protocol (Additional file 4).

For the loci classed as over-amplified by the two-cycle method, the median RMA expression levels were 34 for the one-cycle, 26 for the IVT-E and 385 for the two-cycle. All of the genes had expression values below 250 in the one-cycle and IVT-E data. They can therefore be classed as low expressers (Additional file 5).

Loci classed as under-amplified had median RMA expression values of 417 in the one-cycle data, 404 in the IVT-E data and 62 in the two-cycle data. 16 of the 26 genes had expression values of over 500 in the one-cycle data of either the MS or EZ tissues. The under-amplified genes are generally medium to high expressers, although there are a few genes with low levels of expression in the list (Additional file 5).

### The expression levels of mis-amplified loci in other publically available data sets

To determine whether the mis-amplification of specific probe-sets is a general phenomenon, the expression levels of the genes presented in Additional file 4 were analysed in publicly available *Arabidopsis *root data sets [24,25]. These data sets were selected because the biological experiments most closely match the experiment presented here. Whilst there were lab-to-lab variations in the experimental conditions, all the compared samples were generated from Col-0 WT roots. The experiments presented by Stepanova *et al*. [24] compared WT, *aux1-7 *and *ein2-5 *roots exposed to either air or ethylene, or with and without auxin treatment. The 'air-treated' WT sample in the ethylene experiment and the 'mock WT' in the auxin experiment were used for comparison with our data sets. The Vanneste *et al*. [25] experiments compared gene expression in WT and *slr1 *roots after temporal application of α-naphthaleneacetic acid (NAA). For comparison with our data, we used the data generated from WT roots with no NAA application. Data from MS and EZ tissues was averaged to give an expression level for the one-cycle, two-cycle and IVT-E data for root tips.

To allow for the variance in hybridisation levels in each experiment, median expression values on each array were calculated. The expression of each gene on the array was divided by the median expression value for that array to give a -ised expression value to use for comparison between chips. The results showed that genes in the over-amplified list were on average 19.7 fold higher in the two-cycle data compared to one-cycle or IVT-E data (10.6 fold (SE 2.8) in the Vanneste data, 20.7 fold (SE 5.4) in Stepanova WT 'air' data set, 23.8 fold (SE 6.6) in Stepanova WT 'mock' data set, 16.4 fold (SE 3.8) compared to the one-cycle root tip data and 27.2 fold (SE 6.4) compared to the IVT-E root tip data). Genes identified as under-amplified were, on average, 6.3 fold lower in the two-cycle data set than the other experiments (5.2 fold (SE 1.4) in the Vanneste data, 6.5 fold (SE 1.5) in Stepanova WT 'air', 5.8 fold (SE 1.0) in Stepanova WT 'mock', 7.2 fold (SE 0.5) compared to the one-cycle root tip data and 6.6 fold compared to the IVT-E root tip data) (Additional file 6).

At present, there are no public IVT-E data sets available for Arabidopsis root tissue. Therefore in order to further investigate the mis-amplified probes, the expression of the loci were analysed in publicly available data sets which have utilised two-cycle amplification and compared with data of similar tissue types that have used the one-cycle protocol. Root tissues were analysed by comparing average expression across the root sections presented by Birnbaum *et al*. [4], with the values from the wild-type control samples produced by Stepanova *et al*. [24] (two-cycle and one-cycle labelled samples respectively).

Genes classed as under-amplified were expressed an average of 5.4 fold (SE 1.5) lower in two-cycle amplified root tissue compared to the one-cycle samples (Additional file 7). Of the 26 loci classed as under-amplified, 25 were expressed over 1.5 fold lower in two- vs. one-cycle root tissue (Table 3). Genes classed as over-amplified were expressed an average of 2.1 fold (SE 0.3) higher in two-cycle root tissues compared to one-cycle labelled samples (Additional file 6). 8 of the 9 genes in the list were expressed over 1.5 fold higher in the two-cycle root data compared to the one-cycle data (Table 3).

**Table 3:** The over- and under-amplified loci.

Probe ID	current 1-cyc vs. 2-cyc	current IVT-E vs. 2-cyc	Stepanova vs. Birnbaum	AGI code	Description
247762_at	16.4	39.8	2.4	AT5G59170	Proline-rich family protein.
249552_s_at	19.7	25.9	1.8	AT5G38250 AT5G38240	AT5G38250, serine/threonine protein kinase, putative; AT5G38240, serine/threonine protein kinase, putative.
251127_at	3.4	8.3	2.1	AT5G01080	Beta-galactosidase.
252971_at	10.9	22.5	1.6	AT4G38770	PRP4 (PROLINE-RICH PROTEIN 4).
255138_at	12.5	14.2	1.8	AT4G08380	Proline-rich extensin-like family protein.
262566_at	28.2	63.1	3.0	AT1G34310	ARF12 (AUXIN RESPONSE FACTOR 12); transcription factor.
266152_s_at	14.3	17.2	2.8	AT3G31908 AT3G32377 AT2G12050	AT3G31908, pseudogene, similar to aintegumenta-like protein; AT3G32377, pseudogene, similar to aintegumenta-like protein; AT2G12050, pseudogene, embryogenesis protein-related, similar to BABY BOOM (A. thaliana).
266154_at	39.1	47.1	3.0	AT2G12190	Cytochrome P450, putative.

245513_at	4.4	4.0	3.2	AT4G15780	ATVAMP724 (Arabidopsis thaliana vesicle-associated membrane protein 724).
245665_at	4.8	5.1	2.4	AT1G28250	Similar to hypothetical protein [Oryza sativa (japonica cultivar-group)] (GB:BAC84779.1).
246210_at	8.0	6.9	2.3	AT4G36420	Ribosomal protein L12 family protein.
249583_at	5.4	5.8	1.8	AT5G37770	TCH2 (TOUCH 2); calcium ion binding.
250226_at	7.4	6.6	3.6	AT5G13780	GCN5-related N-acetyltransferase, putative.
250935_at	9.0	8.9	39.7	AT5G03240	UBQ3 (POLYUBIQUITIN 3); protein binding.
253189_at	8.3	6.0	5.8	no_match	No_match.
253464_at	6.8	4.7	3.4	AT4G32030	Unknown protein.
253545_at	5.4	7.4	4.0	AT4G31310	Avirulence-responsive protein-related/avirulence induced gene (AIG) protein-related.
256092_at	9.8	12.4	8.3	AT1G20696	HMGB3 (HIGH MOBILITY GROUP B 3); transcription factor.
256231_at	9.4	9.5	5.3	AT3G12630	Zinc finger (AN1-like) family protein.
258001_at	5.5	6.2	9.8	AT3G28950	Avirulence-responsive protein-related/avirulence induced gene (AIG) protein-related.
258397_at	6.1	6.1	1.9	AT3G15357	Unknown protein.
258958_at	7.0	10.4	4.6	AT3G01390	VMA10 (VACUOLAR MEMBRANE ATPASE 10).
259095_at	5.9	6.2	1.7	AT3G05020	ACP1 (ACYL CARRIER PROTEIN 1).
262295_at	7.7	4.9	4.4	AT1G27650	ATU2AF35A; RNA binding.
263878_s_at	4.7	4.4	3.8	AT3G18140 AT2G22040	AT3G18140, transducin family protein/WD-40 repeat family protein; AT2G22040, transducin family protein/WD-40 repeat family protein.
264566_at	6.8	4.6	5.6	AT1G05270	TraB family protein.
264702_at	5.3	5.3	3.0	AT1G70190	Ribosomal protein L12 family protein.
265103_at	12.9	4.2	3.9	AT1G31070	UDP-N-acetylglucosamine pyrophosphorylase-related.
265443_at	6.4	6.7	3.0	AT2G20750	ATEXPB1 (ARABIDOPSIS THALIANA EXPANSIN B1).
266074_at	5.1	8.1	1.7	AT2G18740	Small nuclear ribonucleoprotein E, putative/snRNP-E, putative/Sm protein E, putative.
266125_at	6.9	6.3	1.8	AT2G45050	Zinc finger (GATA type) family protein.
267064_at	6.8	7.9	2.9	AT2G41110	CAM2 (CALMODULIN-2); calcium ion binding.
AFFX-Athal-GAPDH_5_s_at	13.4	8.3	11.8	AT3G04120	GAPC (GLYCERALDEHYDE-3-PHOSPHATE DEHYDROGENASE C SUBUNIT); glyceraldehyde-3-phosphate dehydrogenase.

Loci classed as over- (above the break line) and under-amplified (below the break line) in both the data from this study and in independent one-cycle vs. two-cycle root data sets. Numbers represent the fold change in the comparison. Where several AGI codes are given for a particular probe ID, more than one transcript is likely to hybridise to the probes

### The effect of two-cycle amplification on the identification of potentially differentially regulated genes

Transcriptomic analyses usually result in the production of lists of genes showing expression changes between samples. These genes are then carried forward for post-genomic analyses, for example, through the analysis of knockout or over-expression lines, or visualisation of expression using reporter constructs.

**Figure 3:**
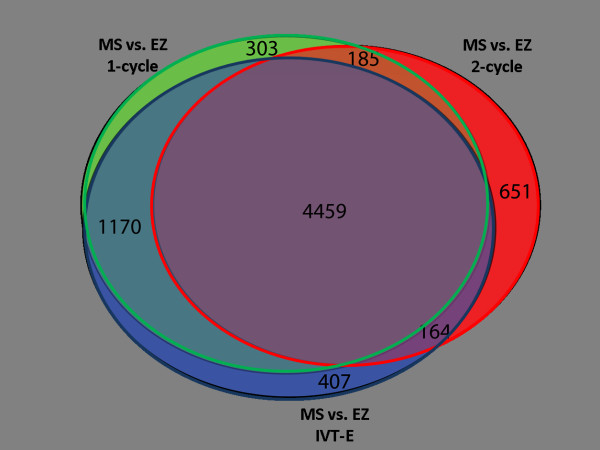
**A Venn diagram of the differentially expressed genes using each protocol**. A proportional Venn diagram showing that lists of differentially regulated loci between MS and EZ tissues created using one- (left circle), two-cycle (right circle) and IVT-E (bottom circle) data sets show a large degree of overlap. Numbers in the segments refer to the number of corresponding loci.

To give an indication of the effect of the different labelling protocols on representation within gene lists, loci that were differentially expressed (DE) between MS and EZ tissues were identified. There were 6117 genes that were classed as DE using the one-cycle data, 5459 genes using the two-cycle data, and 6200 genes using the IVT-E data. 4459 loci were classed as DE with all three protocols (73% of the one-cycle DE genes, 82% of two-cycle DE genes, 72% of the IVT-E DE genes) (Figure 3). Whilst there is a large amount of overlap between the gene lists, this shows that the different labelling protocols have had an effect on the results generated from the experiment, and may lead to altered biological interpretation.

### Bioinformatics analysis of the reason for the mis-amplified probes

In order to investigate the discrepancy in signal between the two-cycle and other protocols for these probesets, several approaches were employed. The first of these was probe-remapping [26], wherein the individual probes for each probeset are re-BLASTED into the current reference build of the Arabidopsis genome held in GenBank. The probe sequences for the ATH1 array were determined in 2001 and in the intervening years continued sequencing and mapping projects have refined and corrected the layout of the genome, the net effect of which is that 11338 probes (4.7%) have moved around the genome and should be associated with other probesets or indeed not included at all. Our hypothesis was that the discrepant genes were biased in the content for probes which were probing for multiple loci, for no loci at all or were otherwise incorrect, leading to a bias in their signals which led to them being more susceptible to amplification effects. Using a remapped chip definition file and reprocessing the data showed that the biases were unaffected by this reanalysis.

A second approach was to examine whether the gene sequences were more or less susceptible to transcriptional and therefore amplificational biases due to self-hybridisation effects. Simple sequence features include high GC content, which would result in higher melting temperatures and potentially reduced transcription rates, and the presence of quadruplexes, which are known to inhibit the progress of complexes along nucleic acids strands [27]. By comparing the sequences of the list of misamplified genes with a randomised selection from the rest of the genome no significant overrepresenation of either GC rich or quadruplex regions was observed.

## Discussion

Transcriptomic analyses have been extensively used within plant sciences in recent years [2,28-33]. Until recently the technique has required a large amount of starting material. This has meant that analysis of small biological samples was prohibited. Two-cycle amplification has allowed small samples to be analysed using microarrays, and has shown that a powerful extra dimension can be added to knowledge by studying only a small subset of cells (for example, [4,7]). Affymetrix has recently introduced the IVT-Express labelling protocol, which can handle both large and small samples.

Whilst various two-cycle labelling protocols have been compared [21,22,34], it has not previously been shown what the effect of two-cycle amplification would have on the representation of plant gene expression in Affymetrix microarray experiments.

We have shown that the Affymetrix one- and two-cycle and IVT-E labelling protocols do not have large effects on the observed gene expression. Lists of DE genes between our two tissue samples were shown to have a large degree of correlation using the one-cycle, two-cycle or IVT-E datasets. The lists, however, were not identical. We have shown that there are a small number of loci that are consistently under- or over-represented on the array after the two-cycle labelling process. There is no obvious mechanistic effect for the misrepresentation of these loci.

It is important to note that many of the probes we have identified as unreliable in two-cycle data relate to genes of biological interest. Included in the lists are at least three genes that encode transcription factors (two zinc-finger proteins and a WD40 repeat family protein), three genes which relate to cellular structural changes (a beta-galactosidase, an extensin and an expansin), and two genes that function in calcium ion binding. An important auxin-related gene is included in the lists (ARF12), highlighting the need for extra caution when looking for responses to auxin with two-cycle data. Of the 33 genes in Additional file 6 and 7, only two are classified as encoding 'unknown proteins' and one has 'no match' to the TAIR 8 genome sequence.

Whilst the lists of loci shown to be mis-represented in different gene lists following two-cycle amplification indicate that data associated with these loci should be treated as uncertain, this list is unlikely to be exhaustive. The data presented here are generated from Arabidopsis root tissue, and we are unable to comment on the behaviour of loci not expressed in our experiments. Therefore, it is likely that there are other probes that have a tendency to be under- or over-amplified using two-cycle labelling.

## Conclusions

We have shown here that two-cycle amplification is an acceptable method of producing transcriptomic data with the Affymetrix ATH1 microarray. One should, however, be as cautious of the differences in the labelling protocols between one- and two-cycle and IVT-E data sets as one would be with any aspect of the transcriptomic workflow [35-38], and refrain from the comparison of absolute expression levels of loci from different protocols. Based on the results presented here, we recommend that extreme caution be placed on the expression level of the highlighted loci when using data sets produced using a two-cycle process. We have found that the Affymetrix IVT-E labelling protocol produces data with less bias than the two-cycle protocol, and as such, would recommend this kit for new experiments that involve small samples.

## Methods

### Biological material

Wild-type Col-0 seedlings were grown vertically on 1/2 MS media [39] supplemented with 1% (w/v) PGP-type agarose (Park Scientific Ltd., UK) for seven days in controlled environmental conditions of 24°C, continuous light of 150 μmol m^-1 ^s^-1^. For each biological replicate, approximately 600 roots were dissected into the meristem (MS) (from the root tip to the top of the lateral root cap, approximately 350 μm from the root tip) and the rapid elongation zone (EZ) (from the top of the lateral root cap to the first visible root hair bulge, approximately 850 μm from the top of the lateral root cap). Dissected samples were immediately frozen in liquid nitrogen. Three biological replicates from separate pools of seeds were used. Plants for seed propagation were grown simultaneous in controlled conditions with a 16 h light (23°C), 8 h dark (18°C) cycle.

### RNA extraction and dilution

RNA was extracted using the Qiagen MicroRNA Kit following the manufacturers recommended protocol (Qiagen, Crawley, UK). RNA was quantified using a Nanodrop ND100 spectrophotometer (Nanodrop, Wilimington, USA). All RNA samples were approximately 1 μg/μl in a total volume of 10 μl. For the two-cycle and IVT-E samples, RNA was diluted with RNase-free water to make a final concentration of 50 ng/μl (equivalent to 30 dissected root sections).

### Amplification and labelling

Labelling of RNA samples was conducted using the Affymetrix One- and Two-Cycle Eukaryotic Target Labelling Assay kits following standard Affymetrix protocols (Affymetrix UK Ltd., High Wycombe, UK). RNA labelling and hybridisation to Affymetrix ATH1 arrays were performed by the Nottingham Arabidopsis Stock Centre (NASC).

### Data analysis

Data were normalised from .cel files using the RMA protocol within R/Bioconductor [40]. Further analyses were performed using Excel 2007 (Microsoft Corporation, Redmond, USA). Comparisons to publicly available data used only wild-type, untreated data sets. Where experiments also contained mutant alleles or hormone treatments, these arrays were excluded from the comparisons. Differentially regulated loci had a fold change greater than 2, and a Benjamini and Hochberg False Discovery Rate of 0.05 (or 5%) [41].

## Accession numbers

Data produced in these experiments have been made available from Arrayexpress http://www.ebi.ac.uk/arrayexpress/ with accession number [E-MEXP-2608]. Other data sets used in this manuscript were obtained from GEO http://www.ncbi.nlm.nih.gov/geo/ with the accession numbers: [GEO: GSE5749] [4], [GEO: GSE432] [24] and [GEO: GSE3350] [25].

## Competing interests

The authors declare that they have no competing interests.

## Authors' contributions

TJH produced the material, generated and analysed the data, and wrote the manuscript. MHW carried out bioinformatic analyses and wrote the manuscript. KK produced the statistical analyses and wrote the manuscript. ILD helped with the statistical analyses and initial direction of the investigation. TCH provided ideas for bioinformatic analyses. ATAW helped with the statistical analyses and the direction for the manuscript. MJH conceived the idea for a manuscript and aided the direction of the analyses. All authors read and approved the final manuscript.

## Supplementary Material

Additional file 1 **Pairwise comparisons of MS and EZ samples using the three different protocols**. Pair-wise comparisons of biological replicates of Log2 data in the meristem (MS, left panels) and elongation zone (EZ, right panels) tissues using the three different labelling protocols - A. 1-cycle; B. 2-cycle; C. IVT-E. R^2 ^values are indicated in each comparison. Click here for file

Additional file 2 **Quantile plots to investigate 5' and 3' bias**. For a given probability *q *between 0 and 1, the *q*-quantile of a data vector is the value *c*_*q*_, such that the proportion of the observations less than *c*_*q *_is equal to *q*. For example, the median is the 0.5-quantile. Some of the biases are very large, so we trim the bias observation vectors and only consider the quantiles between 0.05 and 0.95. These plots are shown above. From the plots, it is clear that the 3' bias is significantly higher for the 2-cycle data. This can be seen by the fact that in both of the plots in the figure, the red lines (representing the two-cycle labelling protocol) are lower than either the black or blue lines (one-cycle and IVT-E protocols). The IVT-E protocol shows marginally more 3' bias than the one-cycle protocol, but this is much less marked a difference than in the case of the two-cycle protocol. Click here for file

Additional file 3 **Loci with absolute residual values greater than 3.5 in pairwise comparisons**. A table showing the ID numbers of probes sets classed as mis-amplified (absolute residual values greater than 3.5) in pairwise comparisons samples using different labelling techniques. The numbers of loci in each list are shown also in Table 2. Click here for file

Additional file 4 **Loci classed as mis-amplified**. A table showing the loci that have been classed as mis-amplified, along with the fold change of the mis-amplification (1-cycle vs. 2-cycle data) for MS and EZ tissues, AGI codes and locus descriptions. Positive fold changes indicate over-amplification, negative fold changes indicate under-amplification. Click here for file

Additional file 5 **RMA expression values of mis-amplified probes**. The RMA normalised expression values of loci identified as mis-amplified in Table 3. Numbers refer to the expression level in the one-cycle, 2-cycle and IVT-E data sets of MS and EZ tissues. Click here for file

Additional file 6 **Relative expression levels of the over- and under-amplified probe sets**. Relative expression levels (compared to median of the array) of the over- (above the break line) and under-amplified (below the break line) probe sets in other plant root data sets. Click here for file

Additional file 7 **Relative expression level of probe IDs classed as over- and under-amplified in publicly available one- and two-cycle data sets**. Relative expression level of probe IDs classed as over- (above the break line) and under-amplified (below the break line) in publicly available one- and two-cycle data sets (Stepanova *et al*. (2007) and Birnbaum *et al*. (2003) respectively). Click here for file
