# Supplementary figures and images for: Statistical evaluation of transcriptomic data generated using the Affymetrix one-cycle, two-cycle and IVT-Express RNA labelling protocols with the Arabidopsis ATH1 microarray
Source: Plant Methods. 2010 Mar 15;6:9. doi: 10.1186/1746-4811-6-9 (PMC2847557; doi:10.1186/1746-4811-6-9)

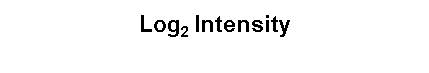

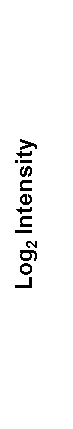

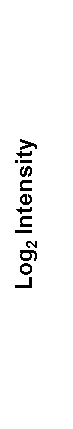

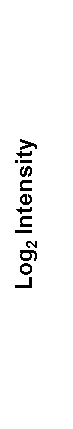

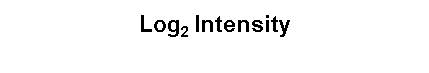
A.
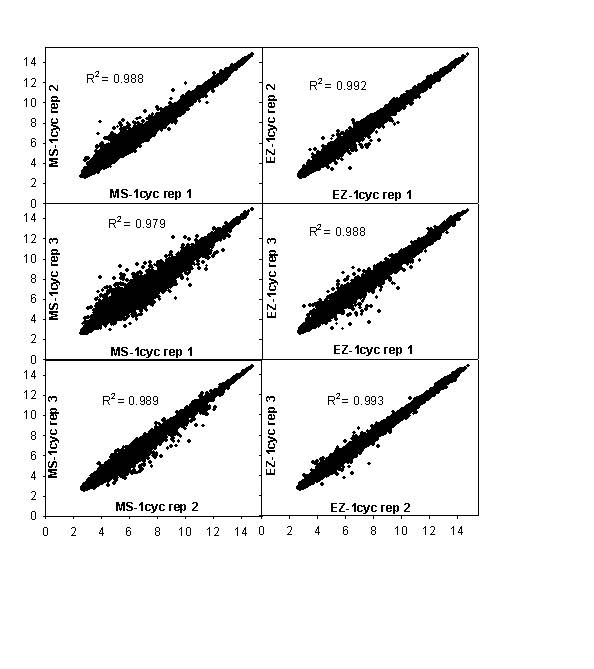


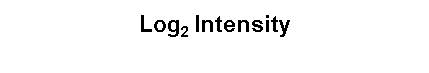

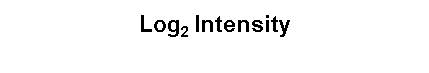

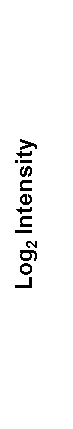

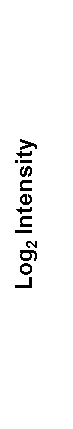

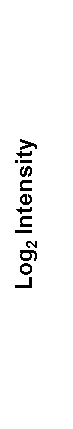
B. *
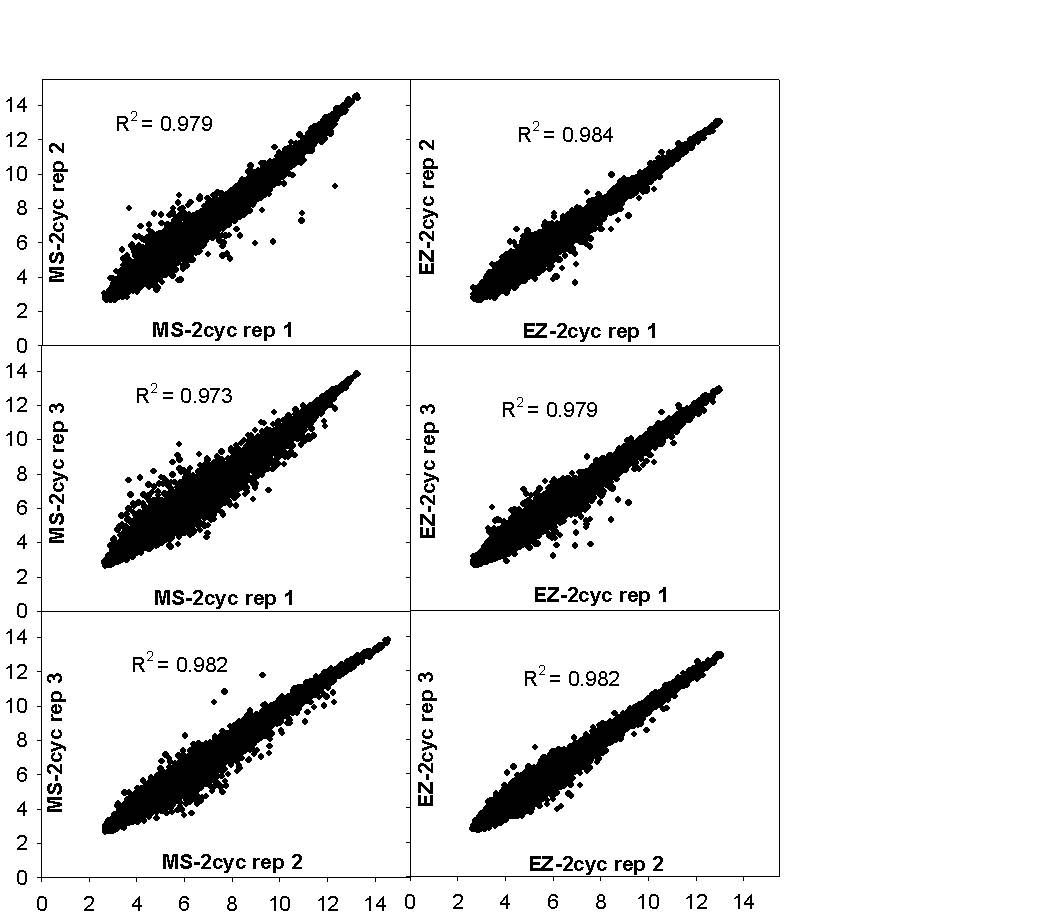
*


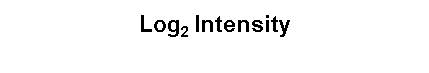

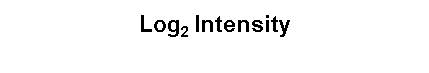

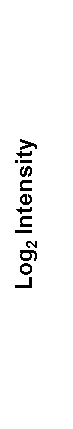

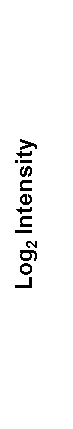

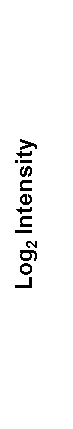

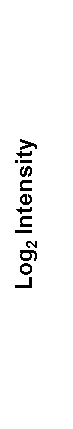
C. *
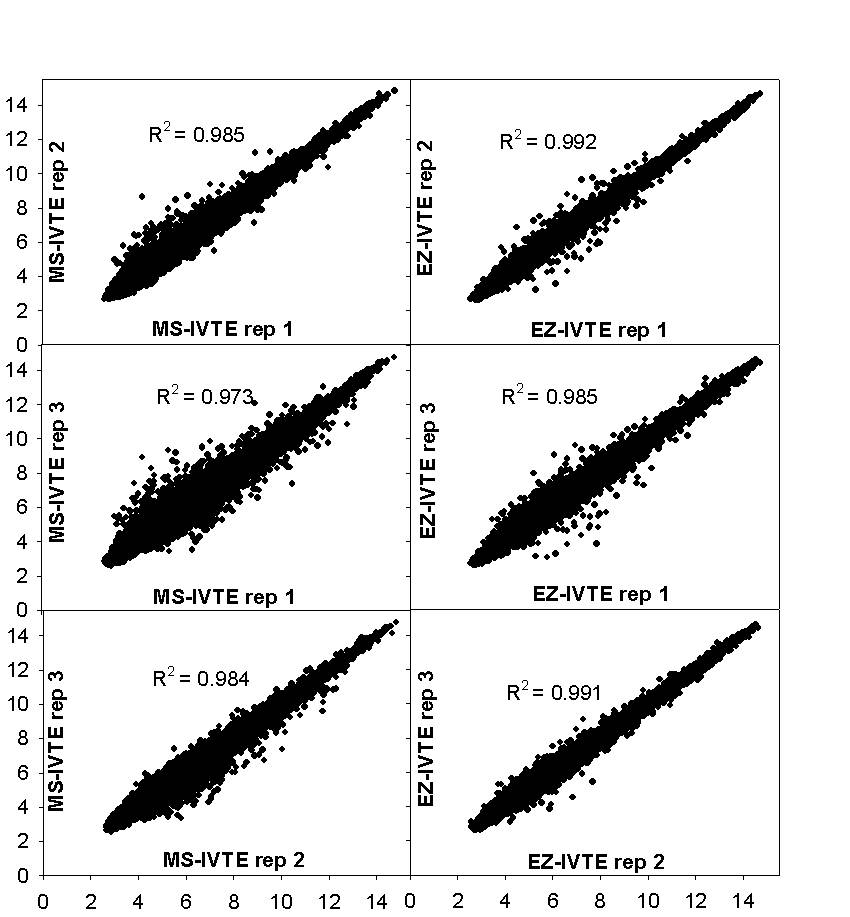
*

Supplement: Additional file 1 — Pairwise comparisons of MS and EZ samples using the three different protocols. Pair-wise comparisons of biological replicates of Log2 data in the meristem (MS, left panels) and elongation zone (EZ, right panels) tissues using the three different labelling protocols - A. 1-cycle; B. 2-cycle; C. IVT-E. R2 values are indicated in each comparison. [file 1746-4811-6-9-S1.DOC]

Meristem

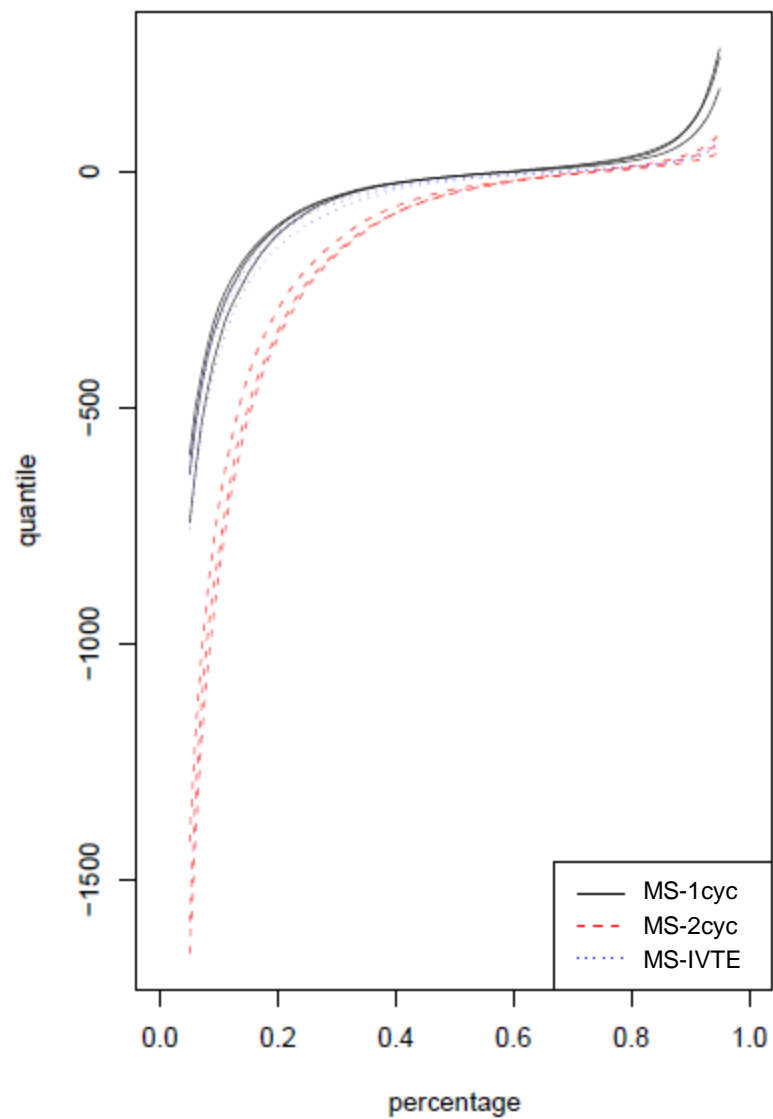

Elongation Zone

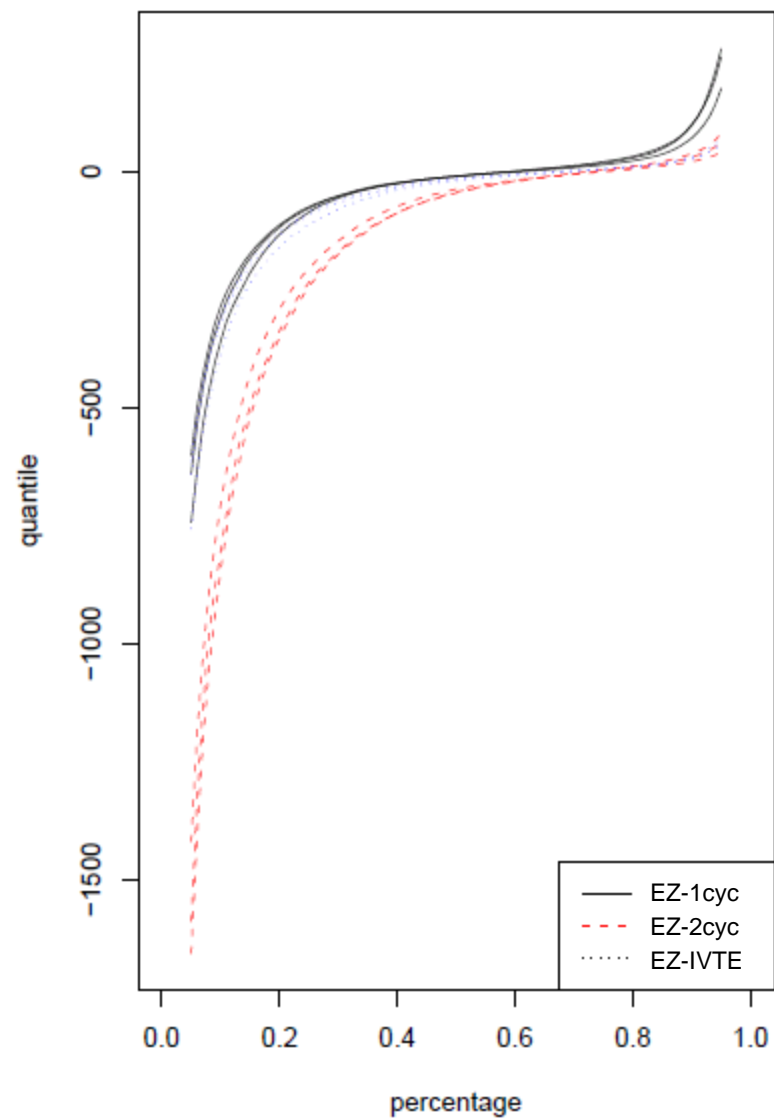

Supplement: Additional file 2 — Quantile plots to investigate 5' and 3' bias. For a given probability q between 0 and 1, the q-quantile of a data vector is the value cq, such that the proportion of the observations less than cq is equal to q. For example, the median is the 0.5-quantile. Some of the biases are very large, so we trim the bias observation vectors and only consider the quantiles between 0.05 and 0.95. These plots are shown above. From the plots, it is clear that the 3' bias is significantly higher for the 2-cycle data. This can be seen by the fact that in both of the plots in the figure, the red lines (representing the two-cycle labelling protocol) are lower than either the black or blue lines (one-cycle and IVT-E protocols). The IVT-E protocol shows marginally more 3' bias than the one-cycle protocol, but this is much less marked a difference than in the case of the two-cycle protocol. [file 1746-4811-6-9-S2.PDF]
