# Supplementary material for: Statistical evaluation of transcriptomic data generated using the Affymetrix one-cycle, two-cycle and IVT-Express RNA labelling protocols with the Arabidopsis ATH1 microarray
Source: Plant Methods. 2010 Mar 15;6:9. doi: 10.1186/1746-4811-6-9 (PMC2847557; doi:10.1186/1746-4811-6-9)
Supplement: Additional file 4 — Loci classed as mis-amplified. A table showing the loci that have been classed as mis-amplified, along with the fold change of the mis-amplification (1-cycle vs. 2-cycle data) for MS and EZ tissues, AGI codes and locus descriptions. Positive fold changes indicate over-amplification, negative fold changes indicate under-amplification. [file 1746-4811-6-9-S4.DOC]

| **Affy ID** | **MS 1-cyc vs. 2-cyc** | **EZ 1-cyc vs. 2-cyc** | **AGI code** | **Description** |
| --- | --- | --- | --- | --- |
| 247762_at | 11.0 | 17.4 | AT5G59170 | Proline-rich family protein |
| 247958_at | 3.7 | 2.3 | AT5G57070 | Hydroxyproline-rich glycoprotein family protein |
| 249552_s_at | 9.1 | 27.3 | AT5G38250 AT5G38240 | AT5G38250, serine/threonine protein kinase, putative AT5G38240, serine/threonine protein kinase, putative |
| 251127_at | 3.3 | 2.9 | AT5G01080 | Beta-galactosidase |
| 252971_at | 5.8 | 13.2 | AT4G38770 | PRP4 (PROLINE-RICH PROTEIN 4) |
| 255138_at | 7.8 | 13.6 | AT4G08380 | Proline-rich extensin-like family protein |
| 262566_at | 20.4 | 29.5 | AT1G34310 | ARF12 (AUXIN RESPONSE FACTOR 12); transcription factor |
| 266152_s_at | 7.8 | 17.0 | AT3G31908 AT3G32377 AT2G12050 | AT3G31908, pseudogene, similar to aintegumenta-like protein {Oryza sativa} AT3G32377, pseudogene, similar to aintegumenta-like protein {Oryza sativa} AT2G12050, pseudogene, embryogenesis protein-related, similar to BABY BOOM (Arabidopsis thaliana). This is very likely a pseudogene. |
| 266154_at | 18.7 | 47.1 | AT2G12190 | Cytochrome P450, putative |
| 244985_at | -6.9 | -9.7 | ATCG00810 | Encodes a chloroplast ribosomal protein L22, a constituent of the large subunit of the ribosomal complex |
| 245513_at | -4.8 | -4.8 | AT4G15780 | ATVAMP724 (Arabidopsis thaliana vesicle-associated membrane protein 724) |
| 245665_at | -4.9 | -5.5 | AT1G28250 | Similar to hypothetical protein [Oryza sativa (japonica cultivar-group)] (GB:BAC84779.1) |
| 246210_at | -8.9 | -8.6 | AT4G36420 | Ribosomal protein L12 family protein |
| 249583_at | -5.1 | -6.7 | AT5G37770 | TCH2 (TOUCH 2); calcium ion binding |
| 250226_at | -8.5 | -7.5 | AT5G13780 | GCN5-related N-acetyltransferase, putative |
| 250935_at | -6.4 | -11.8 | AT5G03240 | UBQ3 (POLYUBIQUITIN 3); protein binding |
| 253189_at | -8.7 | -9.8 | no_match | No_match |
| 253464_at | -6.9 | -8.1 | AT4G32030 | Unknown protein |
| 253545_at | -6.3 | -5.7 | AT4G31310 | Avirulence-responsive protein-related / avirulence induced gene (AIG) protein-related |
| 256092_at | -10.7 | -11.2 | AT1G20696 | HMGB3 (HIGH MOBILITY GROUP B 3); transcription factor |
| 256231_at | -11.3 | -10.1 | AT3G12630 | Zinc finger (AN1-like) family protein |
| 258001_at | -5.6 | -6.6 | AT3G28950 | Avirulence-responsive protein-related / avirulence induced gene (AIG) protein-related |
| 258397_at | -7.2 | -4.3 | AT3G15357 | Unknown protein |
| 258958_at | -6.4 | -8.8 | AT3G01390 | VMA10 (VACUOLAR MEMBRANE ATPASE 10) |
| 259095_at | -5.8 | -7.8 | AT3G05020 | ACP1 (ACYL CARRIER PROTEIN 1) |
| 262295_at | -8.6 | -8.4 | AT1G27650 | ATU2AF35A; RNA binding |
| 263878_s_at | -4.7 | -6.1 | AT3G18140 AT2G22040 | AT3G18140, transducin family protein / WD-40 repeat family protein AT2G22040, transducin family protein / WD-40 repeat family protein |
| 264566_at | -8.4 | -6.8 | AT1G05270 | TraB family protein |
| 264702_at | -5.4 | -7.2 | AT1G70190 | Ribosomal protein L12 family protein |
| 265103_at | -12.3 | -16.6 | AT1G31070 | UDP-N-acetylglucosamine pyrophosphorylase-related |
| 265443_at | -5.1 | -7.7 | AT2G20750 | ATEXPB1 (ARABIDOPSIS THALIANA EXPANSIN B1) |
| 266074_at | -4.9 | -8.8 | AT2G18740 | Small nuclear ribonucleoprotein E, putative / snRNP-E, putative / Sm protein E, putative |
| 266125_at | -8.7 | -6.0 | AT2G45050 | Zinc finger (GATA type) family protein |
| 267064_at | -5.7 | -8.6 | AT2G41110 | CAM2 (CALMODULIN-2); calcium ion binding |
| AFFX-Athal-GAPDH_5_s_at | -13.5 | -16.1 | AT3G04120 | GAPC (GLYCERALDEHYDE-3-PHOSPHATE DEHYDROGENASE C SUBUNIT); glyceraldehyde-3-phosphate dehydrogenase |
