# Supplementary material for: Statistical evaluation of transcriptomic data generated using the Affymetrix one-cycle, two-cycle and IVT-Express RNA labelling protocols with the Arabidopsis ATH1 microarray
Source: Plant Methods. 2010 Mar 15;6:9. doi: 10.1186/1746-4811-6-9 (PMC2847557; doi:10.1186/1746-4811-6-9)
Supplement: Additional file 6 — Relative expression levels of the over- and under-amplified probe sets. Relative expression levels (compared to median of the array) of the over- (above the break line) and under-amplified (below the break line) probe sets in other plant root data sets. [file 1746-4811-6-9-S6.DOC]

| Probe ID | Step AIR | Step MOCK | Van | 1-cycle | 2-cycle | IVT-E | 1-cycle vs. 2-cycle | IVT-E vs. 2-cycle | Step AIR vs. 2-cycle | Step MOCK vs. 2-cycle | Van vs. 2-cycle |
| --- | --- | --- | --- | --- | --- | --- | --- | --- | --- | --- | --- |
| 247762_at | 1.42 | 1.27 | 4.31 | 2.06 | 33.73 | 0.85 | 16.4 | 39.8 | 23.7 | 26.7 | 7.8 |
| 247958_at | 2.60 | 2.09 | 2.25 | 2.45 | 7.27 | 1.16 | 3.0 | 6.3 | 2.8 | 3.5 | 3.2 |
| 249552_s_at | 0.29 | 0.22 | 0.45 | 0.29 | 5.71 | 0.22 | 19.7 | 25.9 | 19.7 | 25.9 | 12.6 |
| 251127_at | 0.81 | 0.76 | 0.96 | 1.25 | 4.29 | 0.52 | 3.4 | 8.3 | 5.3 | 5.6 | 4.5 |
| 252971_at | 0.36 | 0.50 | 0.76 | 0.50 | 5.42 | 0.24 | 10.9 | 22.5 | 15.1 | 10.9 | 7.1 |
| 255138_at | 0.32 | 0.31 | 0.39 | 0.33 | 4.14 | 0.29 | 12.5 | 14.2 | 12.9 | 13.4 | 10.7 |
| 262566_at | 2.21 | 1.88 | 7.34 | 2.51 | 70.78 | 1.12 | 28.2 | 63.1 | 32.0 | 37.6 | 9.6 |
| 266152_s_at | 0.25 | 0.18 | 0.54 | 0.30 | 4.28 | 0.25 | 14.3 | 17.2 | 17.2 | 23.4 | 7.9 |
| 266154_at | 0.34 | 0.29 | 0.62 | 0.50 | 19.68 | 0.42 | 39.1 | 47.1 | 57.2 | 67.6 | 31.5 |
| 244985_at | 2.46 | 3.53 | 8.62 | 14.18 | 2.02 | 10.82 | 7.0 | 5.4 | 1.2 | 1.8 | 4.3 |
| 245513_at | 2.42 | 2.30 | 3.53 | 2.06 | 0.47 | 1.91 | 4.4 | 4.0 | 5.1 | 4.9 | 7.5 |
| 245665_at | 3.64 | 3.53 | 2.56 | 5.00 | 1.04 | 5.33 | 4.8 | 5.1 | 3.5 | 3.4 | 2.5 |
| 246210_at | 2.31 | 2.38 | 0.76 | 6.96 | 0.87 | 5.99 | 8.0 | 6.9 | 2.6 | 2.7 | 0.9 |
| 249583_at | 1.93 | 1.92 | 0.52 | 6.92 | 1.29 | 7.40 | 5.4 | 5.8 | 1.5 | 1.5 | 0.4 |
| 250226_at | 15.49 | 14.00 | 9.97 | 24.74 | 3.33 | 21.99 | 7.4 | 6.6 | 4.6 | 4.2 | 3.0 |
| 250935_at | 46.55 | 28.93 | 35.08 | 10.53 | 1.17 | 10.40 | 9.0 | 8.9 | 39.7 | 24.6 | 29.9 |
| 253189_at | 2.93 | 2.88 | 0.88 | 6.84 | 0.82 | 4.96 | 8.3 | 6.0 | 3.6 | 3.5 | 1.1 |
| 253464_at | 4.68 | 4.04 | 3.91 | 4.79 | 0.70 | 3.29 | 6.8 | 4.7 | 6.6 | 5.7 | 5.6 |
| 253545_at | 3.46 | 2.77 | 2.13 | 2.73 | 0.51 | 3.75 | 5.4 | 7.4 | 6.8 | 5.5 | 4.2 |
| 256092_at | 38.30 | 40.20 | 21.12 | 41.81 | 4.25 | 52.88 | 9.8 | 12.4 | 9.0 | 9.5 | 5.0 |
| 256231_at | 37.25 | 36.31 | 58.50 | 20.71 | 2.19 | 20.81 | 9.4 | 9.5 | 17.0 | 16.5 | 26.7 |
| 258001_at | 11.21 | 10.67 | 6.81 | 5.34 | 0.96 | 5.96 | 5.5 | 6.2 | 11.6 | 11.1 | 7.1 |
| 258397_at | 3.96 | 3.22 | 0.30 | 13.06 | 2.12 | 12.90 | 6.1 | 6.1 | 1.9 | 1.5 | 0.1 |
| 258958_at | 11.48 | 10.93 | 7.82 | 13.41 | 1.92 | 19.99 | 7.0 | 10.4 | 6.0 | 5.7 | 4.1 |
| 259095_at | 11.67 | 10.48 | 10.58 | 27.49 | 4.69 | 29.28 | 5.9 | 6.2 | 2.5 | 2.2 | 2.3 |
| 262295_at | 5.12 | 4.58 | 5.36 | 4.95 | 0.64 | 3.13 | 7.7 | 4.9 | 8.0 | 7.2 | 8.4 |
| 263878_s_at | 2.68 | 2.89 | 1.18 | 2.29 | 0.49 | 2.13 | 4.7 | 4.4 | 5.5 | 5.9 | 2.4 |
| 264566_at | 3.18 | 3.05 | 2.36 | 2.94 | 0.43 | 2.00 | 6.8 | 4.6 | 7.3 | 7.1 | 5.4 |
| 264702_at | 3.17 | 3.22 | 1.20 | 7.52 | 1.43 | 7.63 | 5.3 | 5.3 | 2.2 | 2.3 | 0.8 |
| 265103_at | 1.24 | 1.31 | 1.07 | 4.04 | 0.31 | 1.30 | 12.9 | 4.2 | 4.0 | 4.2 | 3.4 |
| 265443_at | 5.62 | 5.93 | 0.37 | 13.70 | 2.15 | 14.34 | 6.4 | 6.7 | 2.6 | 2.8 | 0.2 |
| 266074_at | 1.93 | 2.33 | 0.60 | 5.59 | 1.10 | 8.94 | 5.1 | 8.1 | 1.8 | 2.1 | 0.5 |
| 266125_at | 1.94 | 1.94 | 0.30 | 6.03 | 0.87 | 5.47 | 6.9 | 6.3 | 2.2 | 2.2 | 0.3 |
| 267064_at | 18.76 | 18.92 | 2.14 | 33.79 | 4.94 | 38.92 | 6.8 | 7.9 | 3.8 | 3.8 | 0.4 |
| AFFX-Athal-GAPDH_5_s_at | 69.39 | 75.27 | 65.76 | 98.62 | 7.35 | 61.11 | 13.4 | 8.3 | 9.4 | 10.2 | 8.9 |
