# Supplementary material for: Statistical evaluation of transcriptomic data generated using the Affymetrix one-cycle, two-cycle and IVT-Express RNA labelling protocols with the Arabidopsis ATH1 microarray
Source: Plant Methods. 2010 Mar 15;6:9. doi: 10.1186/1746-4811-6-9 (PMC2847557; doi:10.1186/1746-4811-6-9)
Supplement: Additional file 7 — Relative expression level of probe IDs classed as over- and under-amplified in publicly available one- and two-cycle data sets. Relative expression level of probe IDs classed as over- (above the break line) and under-amplified (below the break line) in publicly available one- and two-cycle data sets (Stepanova et al. (2007) and Birnbaum et al. (2003) respectively). [file 1746-4811-6-9-S7.DOC]

| Probe ID | Stepanova *et al*. (2007) MOCK | Birnbaum *et al*. (2003) MOCK | Stepanova vs. Birnbaum |
| --- | --- | --- | --- |
| 247762_at | 1.78 | 4.34 | 2.4 |
| 247958_at | 2.13 | 1.42 | 0.7 |
| 249552_s_at | 0.34 | 0.62 | 1.8 |
| 251127_at | 0.69 | 1.44 | 2.1 |
| 252971_at | 0.61 | 0.96 | 1.6 |
| 255138_at | 0.42 | 0.76 | 1.8 |
| 262566_at | 2.53 | 7.67 | 3.0 |
| 266152_s_at | 0.27 | 0.76 | 2.8 |
| 266154_at | 0.35 | 1.05 | 3.0 |
| 244985_at | 3.54 | 5.46 | 0.6 |
| 245513_at | 2.89 | 0.89 | 3.2 |
| 245665_at | 3.58 | 1.50 | 2.4 |
| 246210_at | 2.35 | 1.02 | 2.3 |
| 249583_at | 0.84 | 0.48 | 1.8 |
| 250226_at | 10.73 | 2.96 | 3.6 |
| 250935_at | 8.90 | 0.22 | 39.7 |
| 253189_at | 2.04 | 0.35 | 5.8 |
| 253464_at | 2.34 | 0.69 | 3.4 |
| 253545_at | 2.93 | 0.73 | 4.0 |
| 256092_at | 23.90 | 2.89 | 8.3 |
| 256231_at | 19.30 | 3.67 | 5.3 |
| 258001_at | 12.86 | 1.31 | 9.8 |
| 258397_at | 2.26 | 1.16 | 1.9 |
| 258958_at | 11.23 | 2.45 | 4.6 |
| 259095_at | 6.04 | 3.62 | 1.7 |
| 262295_at | 3.63 | 0.83 | 4.4 |
| 263878_s_at | 2.87 | 0.76 | 3.8 |
| 264566_at | 5.87 | 1.05 | 5.6 |
| 264702_at | 3.21 | 1.08 | 3.0 |
| 265103_at | 0.90 | 0.23 | 3.9 |
| 265443_at | 6.27 | 2.12 | 3.0 |
| 266074_at | 1.56 | 0.90 | 1.7 |
| 266125_at | 2.15 | 1.17 | 1.8 |
| 267064_at | 11.81 | 4.03 | 2.9 |
| AFFX-Athal-GAPDH_5_s_at | 44.16 | 3.75 | 11.8 |
