# Supplementary material for: Statistical evaluation of transcriptomic data generated using the Affymetrix one-cycle, two-cycle and IVT-Express RNA labelling protocols with the Arabidopsis ATH1 microarray
Source: Plant Methods. 2010 Mar 15;6:9. doi: 10.1186/1746-4811-6-9 (PMC2847557; doi:10.1186/1746-4811-6-9)
Supplement: Supplementary file 6 [file 1746-4811-6-9-S5.DOC]

Probe	MS 1-cyc	MS 2-cyc	MS IVT-E	EZ 1-cyc	EZ 2-cyc	EZ IVT-E		247762_at	113	1241	46	144	2508	61		247958_at	74	276	27	230	534	118		249552_s_at	20	181	13	17	452	15		251127_at	84	275	39	72	213	27		252971_at	30	171	14	33	432	16		255138_at	18	143	13	23	318	24		262566_at	150	3068	77	164	4817	65		266152_s_at	17	136	15	20	339	16		266154_at	28	521	26	35	1656	27		244985_at	1135	164	812	652	67	557		245513_at	157	33	147	103	21	95		245665_at	198	40	221	425	77	448		246210_at	671	76	547	210	25	216		249583_at	359	70	360	506	75	574		250226_at	2239	263	1983	888	119	817		250935_at	298	46	284	1008	85	1018		253189_at	451	52	315	408	42	312		253464_at	264	38	191	335	41	225		253545_at	189	30	267	154	27	208		256092_at	3394	317	4141	1879	167	2576		256231_at	927	82	912	1656	164	1708		258001_at	259	47	259	408	62	491		258397_at	1508	211	1463	155	36	190		258958_at	646	101	908	1028	117	1607		259095_at	2166	372	2314	1298	165	1403		262295_at	395	46	239	229	27	159		263878_s_at	180	38	158	109	18	112		264566_at	192	23	120	176	26	131		264702_at	690	129	679	261	36	293		265103_at	227	18	87	279	17	77		265443_at	275	54	278	1421	185	1515		266074_at	509	104	797	198	23	341		266125_at	544	63	494	218	36	201		267064_at	1145	200	1367	3058	355	3519		AFFX-Athal-GAPDH_5_s_at	5088	377	3294	7238	451	4409
